# Sleep spindles across youth affected by schizophrenia or anti-*N*-methyl-D-aspartate-receptor encephalitis

**DOI:** 10.3389/fpsyt.2023.1055459

**Published:** 2023-06-07

**Authors:** Maria E. Dimitriades, Andjela Markovic, Silvano R. Gefferie, Ashura Buckley, David I. Driver, Judith L. Rapoport, Margherita Nosadini, Kevin Rostasy, Stefano Sartori, Agnese Suppiej, Salome Kurth, Maurizia Franscini, Susanne Walitza, Reto Huber, Leila Tarokh, Bigna K. Bölsterli, Miriam Gerstenberg

**Affiliations:** ^1^Child Development Center, University Children's Hospital Zurich, Zurich, Switzerland; ^2^Children's Research Center, University Children's Hospital Zurich, Zurich, Switzerland; ^3^Department of Pulmonology, University Hospital Zurich, Zurich, Switzerland; ^4^University Hospital of Child and Adolescent Psychiatry and Psychotherapy, University of Bern, Bern, Switzerland; ^5^Department of Psychology, University of Fribourg, Fribourg, Switzerland; ^6^Stichting Epilepsie Instellingen Nederland, Heemstede, Netherlands; ^7^Department of Neurology, Leiden University Medical Center, Leiden, Netherlands; ^8^Pediatrics and Neurodevelopmental Neuroscience, National Institute of Mental Health, Bethesda, MD, United States; ^9^Child Psychiatry Branch, National Institute of Mental Health, Bethesda, MD, United States; ^10^Paediatric Neurology and Neurophysiology Unit, Department of Women's and Children's Health, University Hospital of Padova, Padova, Italy; ^11^Neuroimmunology Group, Paediatric Research Institute Città della Speranza, Padova, Italy; ^12^Department of Pediatric Neurology, Children's Hospital Datteln, Witten/Herdecke University, Datteln, Germany; ^13^Department of Medical Sciences, Pediatric Section, University of Ferrara, Ferrara, Italy; ^14^Department of Child and Adolescent Psychiatry and Psychotherapy, Psychiatric University Hospital Zurich, University of Zurich, Zurich, Switzerland; ^15^Neuroscience Center Zurich, University of Zurich and Swiss Federal Institute of Technology Zurich, Zurich, Switzerland; ^16^Zurich Center for Integrative Human Physiology, University of Zurich, Zurich, Switzerland; ^17^Translational Research Center, University Hospital of Psychiatry and Psychotherapy, University of Bern, Bern, Switzerland; ^18^Department of Pediatric Neurology, University Children's Hospital Zurich, Zurich, Switzerland; ^19^Department of Pediatric Neurology, Children's Hospital of Eastern Switzerland, St. Gallen, Switzerland

**Keywords:** psychosis, schizophrenia, anti-NMDAR encephalitis, sleep EEG, sleep spindles, thalamocortical network

## Abstract

**Background:**

Sleep disturbances are intertwined with the progression and pathophysiology of psychotic symptoms in schizophrenia. Reductions in sleep spindles, a major electrophysiological oscillation during non-rapid eye movement sleep, have been identified in patients with schizophrenia as a potential biomarker representing the impaired integrity of the thalamocortical network. Altered glutamatergic neurotransmission within this network via a hypofunction of the *N*-methyl-D-aspartate receptor (NMDAR) is one of the hypotheses at the heart of schizophrenia. This pathomechanism and the symptomatology are shared by anti-NMDAR encephalitis (NMDARE), where antibodies specific to the NMDAR induce a reduction of functional NMDAR. However, sleep spindle parameters have yet to be investigated in NMDARE and a comparison of these rare patients with young individuals with schizophrenia and healthy controls (HC) is lacking. This study aims to assess and compare sleep spindles across young patients affected by Childhood-Onset Schizophrenia (COS), Early-Onset Schizophrenia, (EOS), or NMDARE and HC. Further, the potential relationship between sleep spindle parameters in COS and EOS and the duration of the disease is examined.

**Methods:**

Sleep EEG data of patients with COS (*N* = 17), EOS (*N* = 11), NMDARE (*N* = 8) aged 7–21 years old, and age- and sex-matched HC (*N* = 36) were assessed in 17 (COS, EOS) or 5 (NMDARE) electrodes. Sleep spindle parameters (sleep spindle density, maximum amplitude, and sigma power) were analyzed.

**Results:**

Central sleep spindle density, maximum amplitude, and sigma power were reduced when comparing all patients with psychosis to all HC. Between patient group comparisons showed no differences in central spindle density but lower central maximum amplitude and sigma power in patients with COS compared to patients with EOS or NMDARE. Assessing the topography of spindle density, it was significantly reduced over 15/17 electrodes in COS, 3/17 in EOS, and 0/5 in NMDARE compared to HC. In the pooled sample of COS and EOS, a longer duration of illness was associated with lower central sigma power.

**Conclusions:**

Patients with COS demonstrated more pronounced impairments of sleep spindles compared to patients with EOS and NMDARE. In this sample, there is no strong evidence that changes in NMDAR activity are related to spindle deficits.

## 1. Introduction

Once thought to be merely a phenotype of psychosis, sleep dysfunction is now considered to be intricately intertwined in the pathophysiology of disorders along the psychosis spectrum (1–4). Schizophrenia (SZ) is one of several conditions that falls within the psychosis spectrum and is characterized by psychotic symptoms, marking a disconnection from reality, and considerable changes in affective state, perception, and cognition. Disturbed sleep often precedes the onset of SZ and predicts relapses in remitted patients (5–7). Thus, increased research efforts are made to study sleep quality, structure, and electroencephalographic characteristics in order to identify specific parameters potentially contributing to or resulting from the emergence of the disorder. Specifically sleep spindles, a sleep electroencephalogram (EEG) characteristic, caught much attention as a potential endophenotype as well as a target for novel therapeutic approaches (8). Sleep spindles are major oscillations occurring in the sigma frequency range (12–15 Hz) and are most prominent during stage 2 of Non-Rapid Eye Movement (NREM) sleep (3). The reduction of sleep spindle density (SSD; number per minute) in patients affected by SZ when compared to healthy controls (HC) has been the most consistent finding across studies and diverse patient populations with varying age, medication status, or chronicity of the disorder (3, 8–10). This finding is most pronounced in central regions of the cortex in the fast frequency range of spindles (13–15 Hz) (3, 9, 11). Reductions in sleep spindle maximum amplitude (SSMA) and sigma power (SP) are also frequently reported, but tend to be less consistently reduced across studies. This may be in part due to differences in electrode density montages, detection methods, and chronicity of the disorder (1, 3, 9–11).

Sleep spindles are generated by inhibitory GABAergic (gamma-aminobutyric acid) neurons in the thalamic reticular nucleus (12). Thalamic reticular nucleus neurons inhibit excitatory, glutamatergic neurons that propagate signals from the thalamus to the cortex. Cortical projection neurons further communicate with thalamic reticular nucleus neurons via the activation of specific receptors, including the glutamate-specific ionotrophic receptor, the *N*-methyl-D-aspartate receptor (NMDAR) (13, 14). This NMDAR-mediated glutamatergic feedback loop is necessary to modulate and maintain sleep spindles (15, 16). The NMDAR is also highly implicated in the emergence of SZ. According to the glutamate hypothesis, glutamatergic activity is disrupted in patients with SZ due to a hypofunction of the NMDAR (17, 18). This provokes a cascade of altered neurotransmission that affects brain development and results in the diverse symptomatology of SZ, including cognitive deficits. Sleep spindles play a role in sleep-dependent memory consolidation, and the decrease in sleep spindles in schizophrenia has been hypothesized to contribute to the cognitive deficits that are integral to the disorder (8, 19). It is currently unclear if the hypofunction of the NMDAR is also a key element contributing to the detected sleep spindle reduction in SZ.

A rare form of autoimmune encephalitis, anti-NMDAR encephalitis (NMDARE), is induced by autoantibodies that target the NMDAR leading to a functional deficit (20). The symptomatology of NMDARE and SZ also greatly overlap, often presenting with sleep and cognitive disturbances and culminating with a range of psychotic symptoms (21, 22). This has encouraged research in the context of the glutamate hypothesis of SZ (23–28). Although a previous study found that SSD and SP were only reduced in patients with SZ when comparing them to other patients with mental health disorders who presented with psychosis, sleep spindles have yet to be compared to patient populations with somatic illnesses who present with psychosis (29). The shared phenotype and key pathomechanistic element make SZ and NMDARE very promising to study in concert. Further insight may be gained into the role of the NMDAR within the thalamo-cortical network and the detectable sleep spindle phenotype of both disorders.

Highly dynamic phases of brain development could potentially influence the relationship between sleep and psychosis spectrum disorders. During childhood and adolescence, individuals undergo drastic brain maturation that affects sleep structure and characteristics (25). Additionally, these periods introduce vulnerabilities in the developing cortex which may coincide with the onset of mental health disorders (30, 31). SZ rarely emerges during these young, malleable periods, being classified as Childhood-Onset (COS) if the onset age precedes the age of 13 and Early-Onset Schizophrenia (EOS) if the onset age occurs between age 13 and 18. Previous studies indicate that these populations have reductions in SSD (32, 33), although the two have never been directly compared. In COS, a longer duration of illness was associated with lower SSMA and SP and a recent meta-analysis in adults with SZ points to an association of a longer duration of illness with SSD deficits (33, 34). Combining data from young patients with SZ will allow to further assess the relationship between the duration of illness and sleep spindle parameters.

Pooling sleep data from young patients affected by COS, EOS, and NMDARE, this study aims to assess sleep spindle parameters (SSD, SSMA, SP) between the patient groups and to compare them to age- and sex-matched HC. Due to evidence demonstrating reduced sleep spindles in SZ and the overlapping phenotype and pathomechanism of SZ and NMDARE, it is hypothesized that the pooled patient group will have reduced sleep spindle parameters when compared to HC. Between-patient group comparisons will be performed to elucidate the contribution of each patient population to this presumed finding. Further, to analyze potential regional differences across the cortex, topographical distribution of spindle parameters will be compared between each patient group and matched HC. Lastly, the impact of the duration of illness on spindle alterations will be assessed in an exploratory correlation analysis in young patients with SZ.

## 2. Materials and methods

### 2.1. Participants

Sleep data from three patient groups were gathered for the purpose of this study. Screening procedures and characteristics of patient groups have previously been published (22, 32, 33). All-night EEG, demographic, and clinical data from individuals with COS (*N* = 17, 16.0 ± 3.6 years, 70.6% female), EOS (*N* = 11, 16.4 ± 1.4 years, 36.4% female), NMDARE (*N* = 8, 13.3 ± 4.5 years, 75.0% female), and 36 age- and sex-matched HC were pooled in this project. Patients with SZ (COS, EOS) were either recruited at the National Institute of Mental Health in the United States (COS) or at the Department of Child and Adolescent Psychiatry of the Psychiatric University Hospital Zurich in Switzerland (EOS). The diagnosis for patients with COS was assessed by DSM-III-R or DSM-IV criteria and confirmed by two child psychiatrists after an in-patient stay where medication was discontinued (35, 36). For patients with EOS, mental disorders were assessed by the Mini International Neuropsychiatric Interview for Children and Adolescents (MINI-Kid), and criteria for EOS were further verified according to the DSM-IV (37). Age of onset, duration of illness, and antipsychotic medication measured as chlorpromazine equivalent were provided for each patient with SZ. IQ scores were available from 12 patients with COS and eight with EOS. One individual with EOS declined further use of non-genetic personal health data and was excluded in this study.

In the NMDARE group, three individuals had two EEG recordings available. Sleep structure and spindle parameters were calculated individually for each of these EEG and averaged to have one value for each participant. Patient data for this group was provided by three clinics, specifically the Department of Neuropediatrics, University Children's Hospital Zurich (Switzerland) (*N* = 4; EEG = 6); the Pediatric Neurology and Neurophysiology Unit in Padua (Italy) (*N* = 2; EEG = 3); the Department of Pediatric Neurology, Children's Hospital in Datteln, University Witten/Herdecke (Germany) (*N* = 2; EEG = 2). Clinically suspected diagnosis of NMDARE was confirmed by detection of ANMDAR antibodies in serum and/or cerebrospinal fluid.

The three patient groups had different age and sex characteristics (Table 1). Thirty-six HC were used in total; 17 were age- and sex-matched to the COS group (HC-C), accordingly 11 were matched to the EOS group (HC-E), and eight to the NMDARE group (HC-N). HC were recruited at the University Children's Hospital Zurich and underwent a screening process, excluding individuals with a history of mental health disorders and who took psychotropic medication. Written informed consent was obtained from all participants and/or their legal guardian. This research project was approved by swissethics (BASEC 2021-00215). All study procedures were performed according to the Declaration of Helsinki.

**Table 1:** Demographic and clinical characteristics.

**Parameter**	**Patient group(s) (*N*)**	**Mean (SD)**	**HC: Mean (SD)**	***p*-value**	**Direction (adjusted *p*-value)**
Age (years)	COS (17)	16.00 (3.64)	16.41 (4.01)	0.89	
	EOS (11)	16.38 (1.43)	16.48 (1.71)	0.95	
	NMDARE (8)	13.29 (4.52)	13.65 (4.48)	0.80	
	COS, EOS, NMDARE			0.19	
Sex, female (*n*)	COS (17)	12 (70.59)	12 (70.59)	1.00	
	EOS (11)	4 (36.36)	4 (36.36)	1.00	
	NMDARE (8)	6 (75.00)	6 (75.00)	1.00	
	COS, EOS, NMDARE			0.14	
Age of onset (years)	COS (17)	9.27 (1.79)	NaN (NaN)	NaN	
	EOS (11)	15.33 (1.45)	NaN (NaN)	NaN	
	NMDARE (8)	12.03 (3.46)	NaN (NaN)	NaN	
	COS, EOS, NMDARE			**<0.0001**	COS < EOS (**<0.0001**)
Duration of illness (months)	COS (17)	80.75 (43.51)	NaN (NaN)	NaN	
	EOS (11)	12.56 (11.01)	NaN (NaN)	NaN	
	NMDARE	NaN (NaN)	NaN (NaN)	NaN	
	COS, EOS			**<0.0001**	
Chlorpromazine equivalent (mg)	COS (17)	667.65 (372.05)	NaN (NaN)	NaN	
	EOS (11)	172.50 (158.43)	NaN (NaN)	NaN	
	NMDARE	NaN (NaN)	NaN (NaN)	NaN	
	COS, EOS			**<0.001**	
IQ	COS (12)	78.00 (19.21)	NaN (NaN)	NaN	
	EOS (8)	95.13 (26.44)	NaN (NaN)	NaN	
	NMDARE	NaN (NaN)	NaN (NaN)	NaN	
	COS, EOS			0.13	

COS, childhood-onset schizophrenia; EOS, early-onset schizophrenia; NMDARE, anti-*N*-methyl-D-aspartate receptor encephalitis; SD, standard deviation; NaN, not a number, data missing. HC were 1:1 matched to each patient. When comparing the three patient groups with Kruskal Wallis Tests, any significant difference is reported represented by the adjusted *p*-value and direction of the difference. When comparing only the COS and EOS groups, Mann-Whitney *U*-tests were used. The bold values imply statistical significance.

### 2.2. EEG recordings

All-night polysomnography recordings from patients with COS were measured using a 21-channel 10-20 system [TWin or Nihon Kohden; sampling rate (SR): 200 Hz]. EEG data from patients with EOS and HC were recorded using a 128-channel system (Electrical Geodesic Sensory Net; SR: 500 Hz). Nineteen electrodes were shared between patients with SZ and associated HC: two prefrontal (Fp1, Fp2), three frontal (F3, Fz, F4), three central (C3, C4, Cz; Cz available as a recording electrode following re-referencing), six temporal (F7, F8, T3, T4, T5, T6), three parietal (P3, Pz, P4), and two occipital (O1, O2). The patients with NMDARE had heterogeneous EEG recording protocols: (a) Zurich, 21-channel 10-20 system (NeurofileXP EE; SR: 256 Hz), (b) Padua, 8-10 channels (Galileo NT Software; SR: 256 Hz), and (c) Datteln, 11 channels (Neurofax EEG-9210 System; SR: 200 Hz). Seven electrodes were shared across the EEG montages for all three groups (COS, EOS and NMDARE): two prefrontal (Fp1, Fp2), two temporal (T3, T4), two occipital (O1, O2), and one central (Cz).

Epoch-specific sleep staging and artifact rejection were available from previous analyses (22, 32, 33). Sleep stages were scored based on standard criteria by a sleep expert and either validated by a second sleep expert (COS, EOS, HC) or by one rater only to reduce inter-rater variability (NMDARE) (38). Standard sleep stages were assigned to each epoch, composed of 20-s (EOS, HC, NMDARE) or 30-s (COS) time windows. Specific epochs were excluded on a channel to channel basis from the analyses if high levels of artifacts were found. A semi-automatic procedure was used to detect artifacts based on power thresholds in the muscle frequency band (20–40 Hz) and slow-wave frequency band (0.8–4.6 Hz, COS; 0.75–4.5 Hz, EOS, NMDARE) (39, 40). Channels with artifacts in < 97% (COS, EOS, HC) or 95% (NMDARE) of their epochs were considered for subsequent analyses.

### 2.3. EEG data analyses

EEG data were analyzed in MATLAB 2017b. Data were low-pass filtered (−6 dB cut-off = 40.5 Hz; Finite Impulse Response (FIR) filter), down-sampled to 125 Hz, and high-pass filtered (−6 dB cut-off = 0.38 Hz, Kaiser Window FIR filter). Electrodes were re-referenced to the mean of the occipital channels (O1, O2). The occipital channels were chosen as they are shared by all participants. Further, all other possible channel locations shared by all EEG montages (frontal, central, temporal) have previously shown disturbances in spindles in SZ (8, 32, 41–46).

Sleep spindles were analyzed during the first hour of artifact-free NREM Stage 2 and 3 for each participant. This is considered the most robust part of sleep, with little or no fragmentation. Sleep spindles were automatically detected based on an established amplitude-threshold algorithm (10). EEG signal was band-pass filtered (−6 dB cut-offs: 12.01 and 16.97 Hz, Chebyshev Type II filter) and the average signal amplitude was calculated for each channel. As signal amplitude varies for each electrode, a threshold was set relative to the average signal amplitude for each channel. Therefore, sleep spindles were detected if fluctuations in amplitude for a given channel surpassed an upper threshold of five times the average signal amplitude. The upper threshold value was determined based on previous studies examining young adults and was confirmed as the best representation of spindle detection after visual inspection (47, 48). The beginning and end of each spindle were defined as the instances where the signal surrounding the peak amplitude dipped below a lower threshold of two times the average signal. The lower duration threshold of a spindle was determined to be 0.3 s (49).

Sleep spindle density (SSD), maximum amplitude (SSMA), and sigma power (SP) were investigated in this project. SSD refers to the number of spindles per minute (13.75–14.75 Hz). SSMA refers to the largest amplitude value (positive or negative; μV) of a given spindle and was calculated for every spindle within the frequency range of interest and averaged together. Spectral analysis of EEG signals were performed for each epoch (Fast Fourier transform, 4-s Hanning window, overlapping by 2-s), resulting in a 0.25 Hz frequency resolution. Sigma power (13.75–14.75 Hz; μV^2^) was calculated for each epoch.

### 2.4. Statistical analyses

Sleep structure parameters and demographic and clinical characteristics were compared between patient groups and their associated HC (HC-C, HC-E, HC-N) using non-parametric Mann-Whitney *U*-tests. The same variables were compared between patient groups using non-parametric Kruskal Wallis tests. Multiple comparisons and determining the direction of interaction terms was carried out with Tukey-Kramer *post-hoc* tests for pairwise comparisons. Significant differences were considered with a *p*-value < 0.05. Sleep structure parameter values were calculated for either (1) the first hour of artifact-free NREM Stage 2 and 3 for each participant (the same time frame used in the sleep spindle analysis), or (2) the longest sleep recording period for each participant. The comparison of sleep structure considers the five recording electrodes shared by all groups. It was not possible to calculate sleep latency or efficiency for the NMDARE group as the time when lights were turned off was not documented.

**Figure 1:**
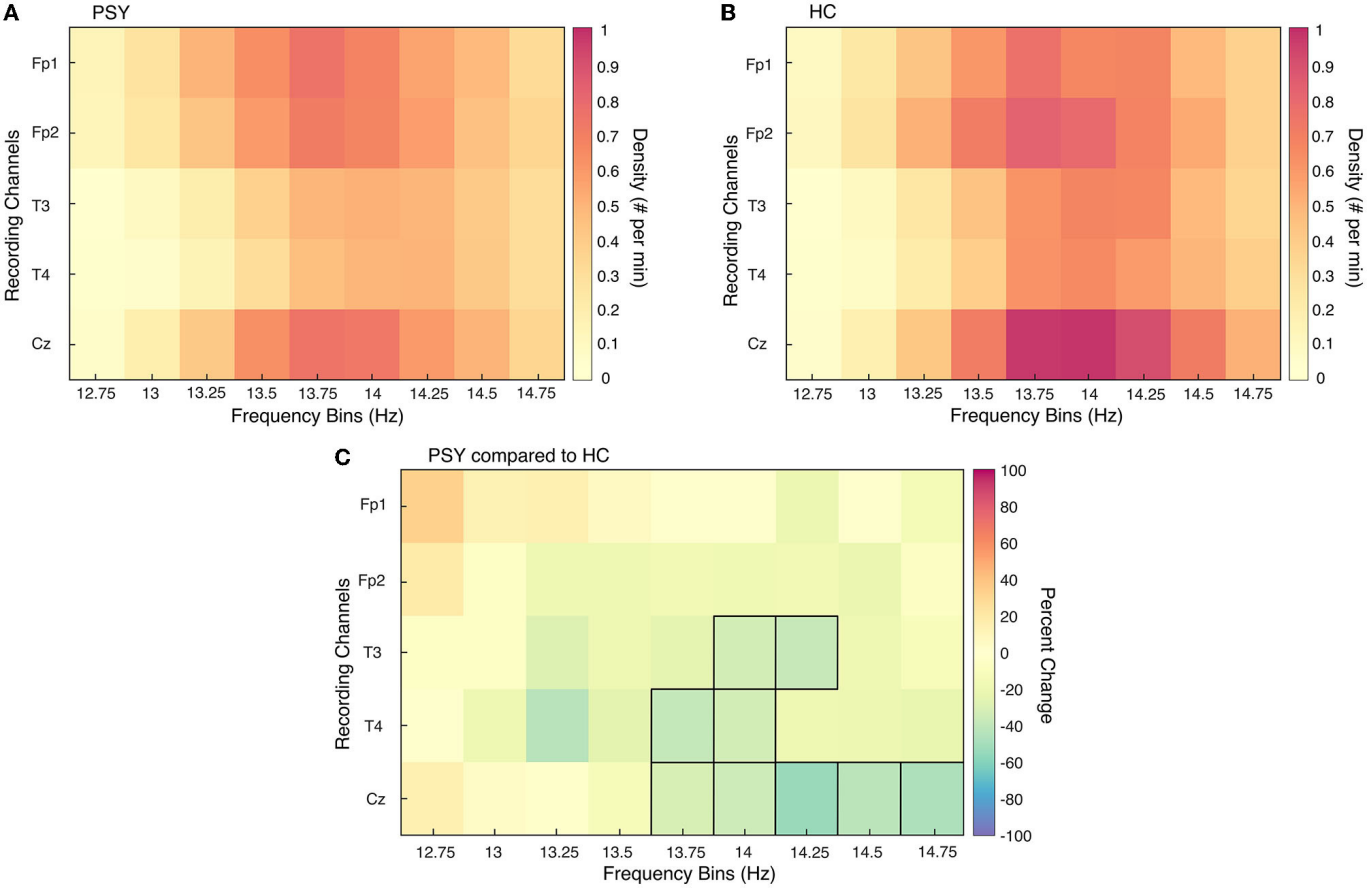
Sleep spindle density differences between PSY and HC for all channels and frequency bins. Sleep spindle density (SSD) is presented for each recording channel for all relevant frequency bins of interest for the collective patient group [PSY; **(A)**] and collective healthy control group [HC; **(B)**]. In **(C)**, the percent change in SSD is directly compared between the two groups; an increase in graph **(C)** represents an increase in SSD in PSY compared to HC. Frequency bin-channel combinations that were significantly different from unpaired *t*-tests between the two groups are outlined in black.

In order to isolate the frequency band and area of interest, the a priori hypothesis regarding SSD in psychosis was investigated. This hypothesis claims that central SSD in the fast spindle frequency range is reduced in patients with psychosis compared to HC. Unpaired *t*-tests assessed SSD differences between the collective patient group (PSY; *N* = 36) and the entire HC sample (*N* = 36). A test was conducted for each recording electrode shared by all participants (Fp1, Fp2, T3, T4, Cz) at each frequency bin of interest (12–15 Hz, discretized into 0.25 steps). Values from the 12–12.5 and 15 Hz frequency bins were excluded from this analysis as the filter attenuated < 20% of the signal amplitude or there were low levels of spindles, respectively. Multiple comparisons were corrected for with Benjamini and Hochberg False Discovery Rate statistical approach. Furthermore, the subsequent spindle analyses focused on values pertaining to the 13.75–14.75 Hz frequency band in the central electrode (Figure 1).

In addition to SSD, SSMA and SP were compared between the PSY and HC groups with unpaired *t*-tests. Differences in spindle parameters between the three patient groups were analyzed with one-way analysis of the variance (ANOVA); Tukey-Kramer *post-hoc* tests were applied subsequently. These two analyses were performed using spindle values specific to the central electrode (Cz). Due to large amounts of artifacts in the central electrode of two patients with NMDARE, they and their age- and sex-matched HC were not included in two above mentioned analyses. Topographical analyses of sleep spindles between each patient group and associated HC were performed with electrode-wise Student's unpaired *t*-tests; multiple comparisons were corrected for with Benjamini and Hochberg False Discovery Rate statistical approach. The comparison considers 17 electrodes for the COS and EOS groups and five electrodes for the NMDARE group. Normal distribution of the data was visualized with quantile-quantile plots and objectively confirmed with Shapiro-Wilk tests.

Furthermore, to assess potential relationships between sleep spindle parameters and illness duration, partial Pearson correlation analysis were performed controlling for age and chlorpromazine equivalent. This analysis was conducted in patients with SZ (COS, EOS). As only patients with SZ were included in this analysis, the value of sleep spindle parameters were taken from the mean of all available central electrodes (C3, Cz, C4).

## 3. Results

### 3.1. Demographic and clinical characteristics and sleep parameters

#### 3.1.1. Demographic and clinical characteristics

No significant difference was found for age and sex between patient groups and associated HC and inter-patient groups (see Table 1). The COS group had a significantly longer duration of illness and a lower age of disease onset when compared to the EOS group. Additionally, the COS group had significantly higher levels of medication, measured in chlorpromazine equivalent, than the EOS group. No significant differences were found in IQ between the COS and EOS groups.

#### 3.1.2. Sleep parameters

Differences in sleep structure and quality for the entire night are reported in Table 2. Patients with COS took longer to fall asleep and spent more time asleep compared to HC-C. After initial sleep onset, they spent less time awake during the entire night. Sleep characteristics were comparable between the patients with EOS and HC-E. Patients with NMDARE slept for significantly longer than HC-N. Differences in sleep parameters were also detected when comparing patient groups. Patients with EOS slept for a shorter amount of time than those with COS. Patients with COS spent less time awake during the entire night following initial sleep onset than those with NMDARE. A statistical difference in percent of all-night time spent in NREM sleep stage 1 was found in the omnibus test, yet no significant differences between the means of each group (COS, EOS, NMDARE) were detected when assessing with *post-hoc* multiple comparisons.

**Table 2:** Sleep structure parameters for the longest sleep recording period.

**Parameter**	**Patient group(s) (*N*)**	**Mean (SD)**	**HC: Mean (SD)**	***p*-value**	**Direction (adjusted *p*-value)**
Sleep latency (min)	COS (17)	36.91 (26.13)	15.47 (7.53)	**0.018**	
	EOS (11)	25.24 (17.24)	17.79 (9.22)	0.38	
	NMDARE	NaN	16.04 (7.95)	NaN	
	COS, EOS, NMDARE			NaN	
Total sleep time (min)	COS (17)	536.38 (168.66)	438.94 (50.74)	**<0.001**	
	EOS (11)	437.85 (123.67)	405.91 (80.39)	0.17	
	NMDARE (8)	557.10 (102.90)	450.58 (43.03)	**0.021**	
	COS, EOS, NMDARE			**0.026**	EOS < COS (**0.04**)
Sleep efficiency (%)	COS (17)	88.23 (17.46)	90.65 (7.51)	1.00	
	EOS (11)	87.91 (11.58)	89.39 (7.702)	1.00	
	NMDARE	NaN	89.68 (5.47)	NaN	
	COS, EOS, NMDARE			NaN	
WASO (min)	COS (17)	28.56 (79.74)	29.51 (33.75)	**0.0079**	
	EOS (11)	27.58 (21.89)	32.58 (31.71)	0.72	
	NMDARE (8)	84.79 (71.31)	38.25 (29.64)	0.20	
	COS, EOS, NMDARE			**0.0024**	COS < NMDARE (**0.0019**)
Sleep stage N1 (%)	COS (17)	3.63 (2.74)	6.54 (5.083)	**0.021**	
	EOS (11)	6.71 (3.93)	6.88 (5.51)	0.84	
	NMDARE (8)	19.04 (25.16)	4.13 (3.02)	0.11	
	COS, EOS, NMDARE			**0.028**	Inconclusive
Sleep stage N2 (%)	COS (17)	49.48 (21.61)	51.50 (6.65)	0.86	
	EOS (11)	48.06 (10.24)	53.04 (3.80)	0.36	
	NMDARE (8)	40.78 (14.88)	47.38 (8.00)	0.51	
	COS, EOS, NMDARE			0.45	
Sleep stage N3 (%)	COS (17)	29.70 (24.46)	23.77 (7.51)	0.95	
	EOS (11)	26.07 (13.01)	22.29 (6.26)	0.89	
	NMDARE (8)	21.8 (9.25)	30.82 (8.32)	**0.083**	
	COS, EOS, NMDARE			0.91	
REM sleep (%)	COS (17)	17.19 (10.73)	18.19 (5.19)	0.78	
	EOS (11)	19.16 (7.69)	17.78 (3.46)	0.29	
	NMDARE (8)	18.38 (9.50)	17.67 (3.27)	0.33	
	COS, EOS, NMDARE			0.66	

COS, childhood-onset schizophrenia; EOS, early-onset schizophrenia; NMDARE, anti-*N*-methyl-D-aspartate receptor encephalitis; WASO, wake after sleep onset; SD, standard deviation; NaN, not a number, data missing. HC were 1:1 matched to each patient. When comparing the three patient groups with Kruskal Wallis Tests, any significant difference is reported represented by the adjusted *p*-value and direction of the difference. The bold values imply statistical significance.

Sleep parameters were also calculated for the time frame used in the sleep spindle analysis (Table 3). This refers to the amount of time it took to isolate 60 minu of artifact-free NREM stage 2 and 3 sleep from each participant. This amount of time was shorter for patients with COS compared to HC-C. Patients with COS also spent less time awake following initial descent into sleep and time in NREM sleep stage 1 during the first hour. The amount of time needed to isolate 60 min of artifact-free sleep in the NMDARE group was significantly longer as when compared to HC-N. Additionally, they spent more time awake and in REM sleep during the first hour compared to HC-N. When comparing patient groups, it was found that the absolute length of the first hour was less in COS when compared to NMDARE. Additionally, the COS group spent less time awake and in NREM sleep stage 1 compared to the EOS and NMDARE group. Finally, both the COS and EOS groups spent less time than the NMDARE group in REM sleep.

**Table 3:** Sleep structure parameters for the first hour of artifact-free NREM stage 2 and 3 sleep.

**Parameter**	**Patient group(s) (*N*)**	**Mean (SD)**	**HC: Mean (SD)**	***p*-value**	**Direction (adjusted *p*-value)**
Absolute length of first hour (min)	COS (17)	70.68 (26.35)	81.12 (38.98)	**0.0071**	
	EOS (11)	74.91 (17.80)	67.49 (2.96)	0.77	
	NMDARE (8)	123 (62.76)	68.25 (5.97)	**0.027**	
	COS, EOS, NMDARE			**0.0028**	COS < NMDARE (**0.0031**)
WASO (%)	COS (17)	0.14 (0.30)	5.01 (11.85)	**0.0019**	
	EOS (11)	5.90 (11.33)	1.43 (2.42)	0.50	
	NMDARE (8)	16.81 (15.73)	2.04 (3.77)	**0.015**	
	COS, EOS, NMDARE			**<0.001**	COS < EOS (**0.045**)
					COS < NMDARE (**<0.001**)
Sleep stage N1 (%)	COS (17)	0.35 (0.70)	4.39 (6.61)	**<0.001**	
	EOS (11)	4.20 (4.78)	2.23 (2.53)	0.45	
	NMDARE (8)	10.62 (15.82)	2.06 (4.21)	**0.063**	
	COS, EOS, NMDARE			**<0.001**	COS < EOS (**0.013**)
					COS < NMDARE (**<0.001**)
Sleep stage N2 (%)	COS (17)	39.04 (27.78)	29.67 (7.05)	0.84	
	EOS (11)	27.13 (8.78)	39.58 (14.74)	0.057	
	NMDARE (8)	27.02 (17.80)	24.97 (24.60)	0.51	
	COS, EOS, NMDARE			0.65	
Sleep stage N3 (%)	COS (17)	57.47 (31.60)	58.49 (21.91)	0.65	
	EOS (11)	62.26 (20.51)	56.45 (15.38)	0.43	
	NMDARE (8)	39.11 (22.01)	70.92 (29.80)	0.051	
	COS, EOS, NMDARE			0.18	
REM sleep (%)	COS (17)	2.95 (8.83)	2.44 (4.64)	0.49	
	EOS (11)	0.50 (1.24)	0.31 (0.78)	0.96	
	NMDARE (8)	6.43 (4.15)	0 (0)	**0.007**	
	COS, EOS, NMDARE			**0.0062**	COS < NMDARE (**0.012**)
					EOS < NMDARE (**0.011**)

COS, childhood-onset schizophrenia; EOS, early-onset schizophrenia; NMDARE, anti-*N*-methyl-D-aspartate receptor encephalitis; WASO, wake after sleep onset; SD, standard deviation. HC were 1:1 matched to each patient. When comparing the three patient groups with Kruskal Wallis Tests, any significant difference is reported represented by the adjusted *p*-value and direction of the difference. The bold values imply statistical significance.

### 3.2. Sleep spindle parameters

#### 3.2.1. Sleep spindle differences between PSY and HC and patient groups

**Figure 2:**
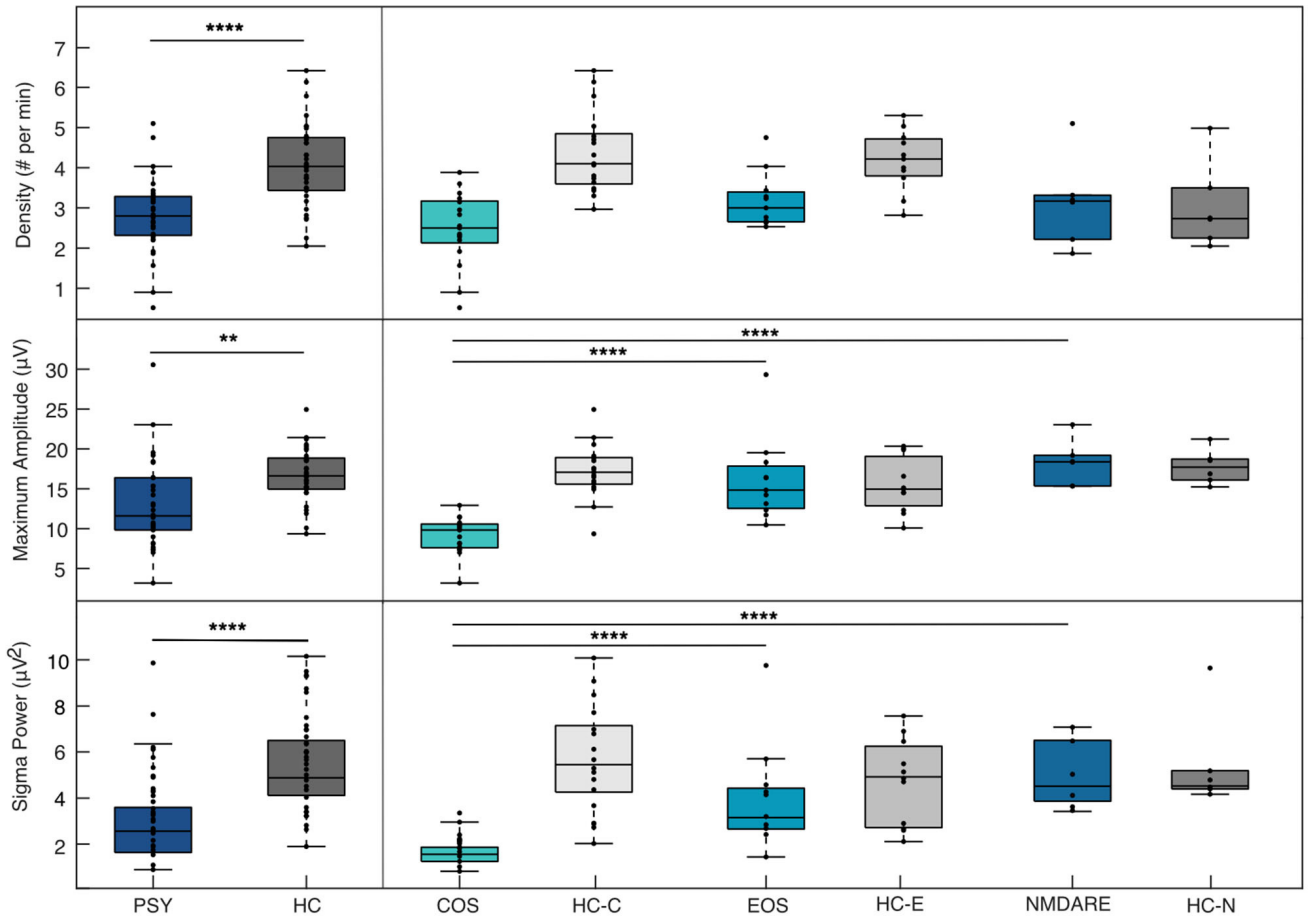
Sleep spindle parameter differences between PSY and HC & patient groups. Differences in sleep spindle parameters between the collective patient groups (PSY) and collective healthy control group (HC) are show in the left panel and between individual patient groups composing the PSY group [Childhood-Onset Schizophrenia (COS), Early-Onset Schizophrenia (EOS), anti-N-methyl-D-aspartate receptor encephalitis (NMDARE)] in the right panel. Note that significant differences between individual patient groups and associated HC are not presented here. All spindle values here are taken from the central electrode from the frequency band of 13.75–14.75 Hz. The * denotes a significant difference between the groups according to *p*-values (< 0.05 implies significance). ^**^ = *p* ≤ 0.01; ^****^ = *p* ≤ 0.0001.

Based on results from the unpaired *t*-test, sleep spindle parameter values from the frequency range of 13.75–14.5 Hz pertaining to the Cz electrode were used to compare the PSY and HC groups and the three patient groups. Results assessing SSD, SSMA, and SP differences between the PSY and HC groups are presented in the left column of Figure 2. For all parameters, the PSY group showed significant reductions in spindle characteristics when compared to the HC group [SSD: *t*(66) = −5.24, *p* < 0.00001; SSMA: *t*(66) = −3.43, *p* = 0.0011; SP: *t*(66) = −4.20, *p* < 0.0001].

ANOVA conducted comparing the three groups and SSD, SSMA, and SP are presented in the right column of Figure 2. No differences in SSD were found between the three groups [*F*(2, 31) = 2.59, *p* = 0.091]. For SSMA and SP, the COS group was significantly reduced compared to the EOS [SSMA: *p* < 0.0001, 95% C.I. = (−10.58, −3.49); SP: *p* < 0.001, 95% C.I. = (−4.03, −1.06)] and the NMDARE groups [SSMA: *p* < 0.0001, 95% C.I. = (−13.49, −4.79); SP: *p* < 0.001, 95% C.I. = (−4.94, −1.29)]. No significant differences in SSMA [*F*(2, 31) = 19.19, *p* = 0.51] and SP [*F*(2, 31) = 13.4, *p* = 0.75] were found between the EOS and NMDARE groups.

#### 3.2.2. Topographical distribution of sleep spindles between patient groups and associated HC

**Figure 3:**
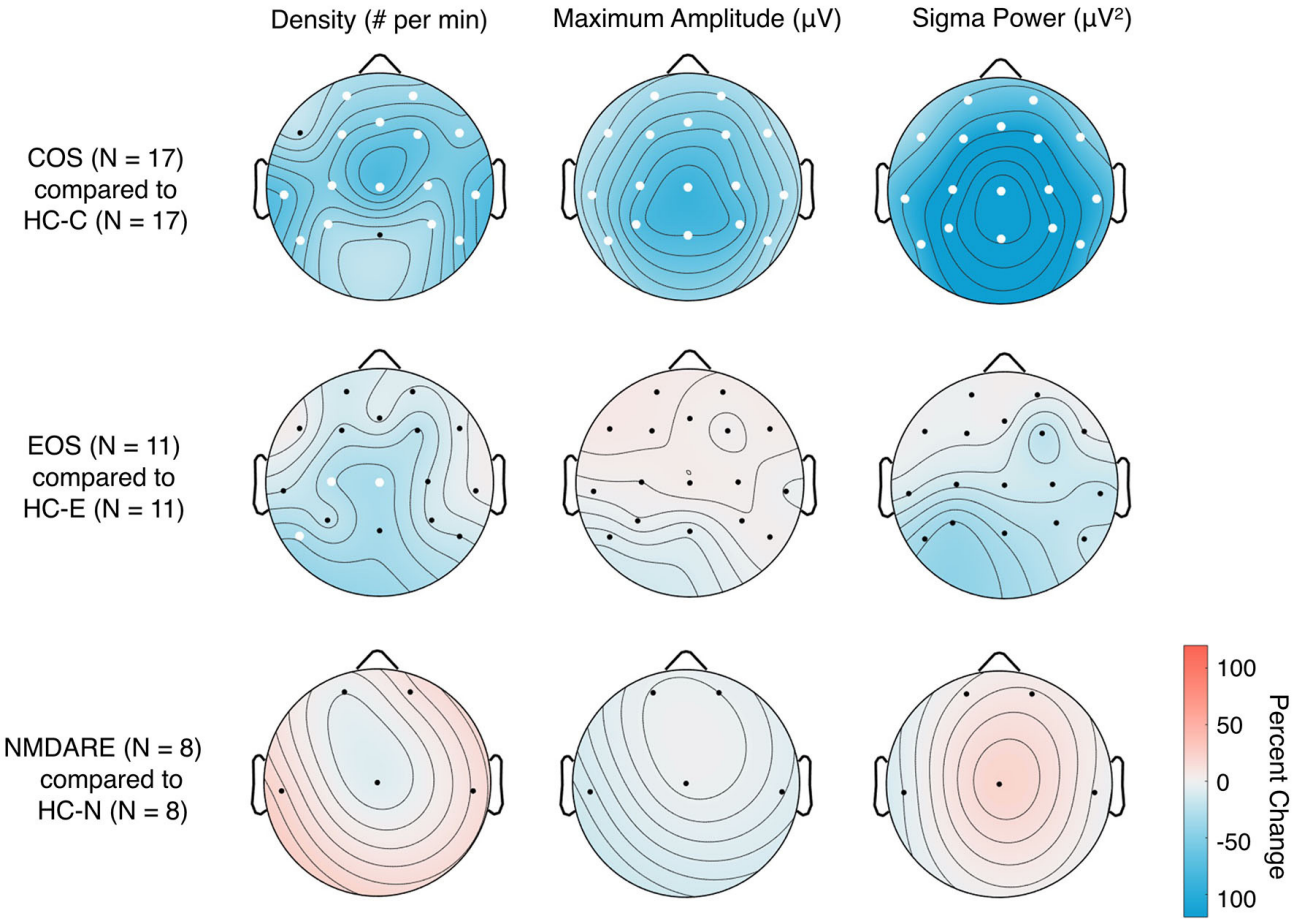
Topographical distribution of sleep spindle parameters in patient groups compared to associated HC. Two-tailed Student's unpaired *t*-tests with standard alpha-values at 0.05 were used; significant differences between the groups in individual electrodes are designated by white dots. The colors represent the percent change of the given sleep spindle parameter when comparing the patient group [Childhood-Onset Schizophrenia (COS), Early-Onset Schizophrenia (EOS), and anti-*N*-methyl-D-aspartate receptor encephalitis (NMDARE)] to the associated healthy control groups (HC-C, HC-E, and HC-N, associated with COS, EOS and NMDARE, respectively) group; e.g., a percent increase represents values that are higher in the patient group compared to HC and a percent decrease represents values that are lower in the patient group compared to HC.

Topographical comparisons of sleep spindle parameters between patient groups and associated HC are presented in Figure 3. As previously reported (33), the COS group shows global significant decreases in all sleep spindle parameters compared to HC-C. As expected, the EOS group shows significant reductions in SSD in the central region and left, posterior temporal region compared to HC-E (32). Sleep spindles for the NMDARE group were statistically comparable to the HC-N group.

### 3.3. Association between sleep spindles and duration of the disorder

**Figure 4:**
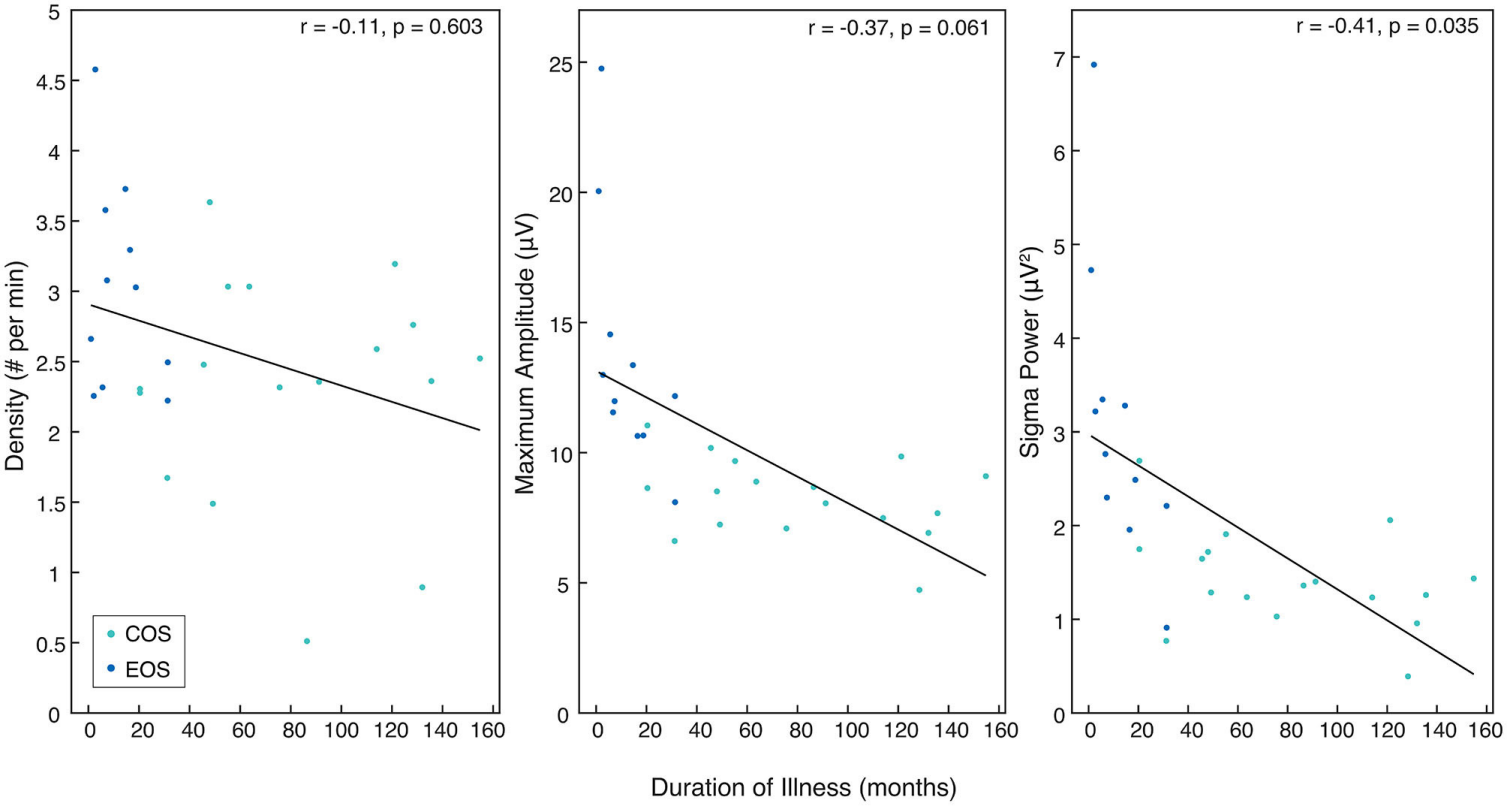
The relationship between sleep spindle parameters in patients with SZ and duration of illness. Rho and *p*-values are presented for each sleep spindle parameter when compared to duration of illness in months. All values come from the mean of the central electrodes (C3, C4, Cz) and pertain to either the Childhood-Onset Schizophrenia (COS) group (light blue) or the Early-Onset Schizophrenia (EOS) group (dark blue).

For all patients with SZ, SP derived from the mean of central electrodes (C3, Cz, C4) was found to be significantly negatively correlated with the duration of illness (*r* = −0.41, *p* = 0.035). Duration of illness was not significantly associated with SSD (*r* = −0.11, *p* = 0.603). There was a trend for a negative correlation between duration of illness and SSMA (*r* = −0.37, *p* = 0.061). All sleep spindle parameters and duration of illness are presented in Figure 4.

## 4. Discussion

The main finding of this study is that all sleep spindle parameters (SSD, SSMA, SP) were reduced in the pooled sample of patients affected by SZ or NMDARE compared to matched HC. SSD was statistically comparable between the three patient groups, however patients with NMDARE showed no significant alterations in sleep spindle parameters when compared to HC-N. Young patients affected by SZ showed reductions in SSD compared to HC; the COS group also exhibited reductions in SSMA and SP. Further, in patients with SZ, reduced SP was associated with a longer duration of illness.

### 4.1. Sleep spindle parameters in patients with SZ

Consistent with previous literature, reduced SSD was found in patients with SZ compared to HC (9, 29, 32, 33). Topographical analysis of SSD indicated widespread reductions in patients with COS, whereas deficits in patients with EOS were restricted to primarily central electrodes. No significant differences were found in SSD measured from the central electrode between the COS and EOS groups. Patients with COS also showed global reductions in SSMA and SP, whereas no changes in these spindle parameters were found in the EOS group when comparing to HC. The COS and EOS groups differ by definition in the age of disease onset, and in this study the COS group also had a longer duration of illness and higher chlorpromazine equivalent than the EOS group. The longer duration of illness is explained by the fact that sleep data from patients with EOS were recorded promptly following disease onset, whereas data from patients with COS were recorded during follow-up assessments years after the initial emergence of the disorder. This is further illustrated by the fact that the mean age at the time of the sleep assessment did not differ between the groups. Thus, in terms of duration of illness, patients with EOS rather resemble adult samples with early course psychosis or first episode SZ whereas young patients with COS may rather be compared to patients with a chronic course of SZ. Considered in this light, the findings are in line with previous literature, namely the vast reduction of SSD in COS corresponds to global reduced SSD in adults with chronic SZ (10, 50). Likewise, the local reduction of SSD in patients with EOS compared to HC-E is consistent with findings in adult patients experiencing their first episode of psychosis (51). Additionally, SSMA and SP reductions in patients with COS are similar to findings from adult samples with chronic SZ (11). When comparing adult patients with chronic SZ to HC, the effect size of the reduction was highest for SSD, followed by SP, and then SSMA. In contrast, in adult first episode psychosis or early course SZ patients there was no significant reduction in SSMA, similar to the results regarding the EOS group (29, 51). Many factors influence spindle characteristics, including genes (52), illness duration (34, 43), and severity of symptomatology (42). Given the current sample size and heterogeneity in our population, we were unable to individually examine the impact of these parameters on spindle characteristics, however, this may be a fruitful future avenue.

The potential impact of the duration of illness in the COS and EOS groups was further explored, resulting in a significant relationship between reduced SP and longer duration of illness. Although individual studies have reported no association between sleep spindle activity and duration of illness or chlorpromazine equivalent in their samples (10, 53), a recent meta-analysis including eight studies demonstrated that across all individuals, the longer the illness lasted, the lower the SSD (34). Specifically for every year of illness, SSD decreased by 0.2 spindles per minute. In conclusion, it is assumed that in this sample, as in previous single studies with similar numbers of patients, the correlation analysis of SSD with duration of illness may have been underpowered. Additionally, while age-specific aspects of sleep spindles may be accounted for by age- and sex-matching when comparing patients and HC, directly comparing the patient sample may be confounded by developmental aspects of sleep spindles across the whole age-range (14, 54).

### 4.2. Sleep spindle parameters in young patients with NMDARE

Central SSD was reduced in the collective patient group compared to HC and no significant differences were found between the patient groups. This suggests that all patient groups contribute to the low, central SSD. However, no significant differences in any of the sleep spindle parameters were found when comparing NMDARE to HC-N. This finding may at least in part be due to the sample size and inter-individual variability making any definite conclusions challenging. Nonetheless, strong and global deficits in sleep spindles as found in patients with COS might have been detected irrespective of these limitations.

The exact molecular mechanism and pathways of neurotransmission underlying spindle deficits in patients with SZ is unclear. It has been suggested that a reduced binding or expression of the glutamatergic NMDAR within the thalamo-cortical system may be the key element contributing to early reductions in SSD (51). Further, postmortem studies found reductions of the NMDAR in the thalamus and in the cortex of patients with SZ (55). On the other hand, based on the premise that the spindle-pacemaker relies on GABAergic neurons, it has been hypothesized that GABA deficits may trigger the reduction in SSD whereas a hypofunction of the NMDAR within the thalamo-cortical feedback loop may rather impact the maintenance and modulation of sleep spindles and their coordination with other oscillations (53). Accordingly, the administration of eszopiclone, a GABA agonist targeting the thalamic reticular nucleus, in patients affected by SZ results in increased SSD (56). Additionally, an *in vitro* model showed that a blockade of the NMDAR shortened the spindle-like oscillation, but only the combined blockade of the NMDAR and GABA receptor repressed the spindle-like oscillation (13). Thus, the findings suggesting that patients with NMDARE do not have large spindle deficits rather support previous literature and the hypothesis that NMDAR-mediated hypofunction alone does not account for pronounced deficits in SSD. Specifically, the explicit antibody-mediated reduction of functional NMDAR in NMDARE may not entirely mimic the far less clear beginning and timing of abnormalities on the NMDAR during development and its impact on GABAergic activity and thalamocortical integrity in SZ (57, 58). Although speculative, it is possible that further research in NMDARE with increased sample size, age range, and density of electrodes may uncover more subtle reductions of SSD, perhaps making it similar to early stages of SZ such as the patient group with EOS.

### 4.3. Limitations and outlook

The results of the present study should be interpreted within the confines of several limitations. The rarity of NMDARE and Childhood- and Early-Onset SZ make the data of the included individuals very valuable, but the low sample size renders the statistical analyses less robust and age- and sex-matching between the patient groups challenging. Additionally, sleep disorders in the NMDARE group of patients were not assessed systematically. Thus, it remains difficult to discern and weigh the impact of the sleep spindle parameter findings, as well as the relationship with duration of illness. Further, analysis of the sleep structure showed group-specific differences that may be due to different medication and recording settings. However, sleep spindles predominantly appear in sleep stage N2, which was comparable between all groups.

Assessing sleep spindles and their coordination with other oscillations, specifically slow waves, in longitudinal studies may be essential to further discern the pathomechanism of SZ and to identify targets for novel interventions to treat this debilitating disorder. Likewise, sleep studies in larger samples of patients with NMDARE or with pharmacological models mimicking the NMDAR hypofunction may further elucidate similarities and dissimilarities of underlying signaling and thalamo-cortical impairments.

## Data availability statement

The original contributions presented in the study are included in the article/supplementary material, further inquiries can be directed to the corresponding author.

## Ethics statement

The studies involving human participants were reviewed and approved by Ethics Committee of the Canton of Zurich and Bern, Switzerland, BASEC 2021-00215. Written informed consent to participate in this study was provided by the participants' legal guardian/next of kin.

## Author contributions

AM, SG, MN, KR, SS, AS, SK, BB, and MG previously collected, provided, and/or analyzed patient data. BB and MG contributed to the conception of the project and the study design. MG additionally contributed to the writing process. MD contributed to the project design, data analysis, and writing process. MG, BB, LT, and RH closely supervised the project. All authors reviewed and approved the article for submission.
